# Acute Effects of Different Melatonin Doses on Performance and Psychophysiological Responses During Exhaustive Cycling Exercise: A Double-Blind Crossover Study

**DOI:** 10.3390/nu18050798

**Published:** 2026-02-28

**Authors:** Larissa de Castro Pedroso, Maria Clara dos Reis, Vanessa Bertolucci, Luana Alves Silva, Ivan Gustavo Masselli dos Reis, Wladimir Rafael Beck, Pedro Paulo Menezes Scariot, Leonardo Henrique Dalcheco Messias

**Affiliations:** 1 Research Group on Technology Applied to Exercise Physiology—GTAFE, Health Sciences and Health Data Science Postgraduate Programs, São Francisco University (USF), Bragança Paulista 12916-900, SP, Brazil; larissa.castro@mail.usf.edu.br (L.d.C.P.); maria.clara.reis@mail.usf.edu.br (M.C.d.R.); vanessa.bertolucci@mail.usf.edu.br (V.B.); silva.alves.luana@mail.usf.edu.br (L.A.S.); ivan.reis@usf.edu.br (I.G.M.d.R.); pedro.scariot@usf.edu.br (P.P.M.S.); 2 Laboratory of Endocrine Physiology and Physical Exercise, Department of Physiological Sciences, Federal University of São Carlos, Washington Luis, Km 235, São Carlos 13565-905, SP, Brazil; beckwr@ufscar.br

**Keywords:** melatonin, exercise, anaerobic threshold, ergogenic aid

## Abstract

**Background/Objectives**: This study examined the acute effects of different doses of melatonin on performance, physiological, and psychophysiological responses during individualized exhaustive cycling exercise. **Methods**: Fifteen physically active but cycling-inexperienced men (18–35 years) completed a double-blind, randomized, placebo-controlled, crossover protocol. Following an incremental test to determine the anaerobic threshold (AnT), participants performed four exhaustive exercise sessions at 80% of AnT after ingesting placebo or melatonin (5, 12.5, or 20 mg), administered approximately 30 min before exercise. Time to exhaustion (TLim) was considered the primary performance outcome. Heart rate, peripheral oxygen saturation, blood lactate concentration, blood glucose, and ratings of perceived exertion were assessed before, during, and after exercise. **Results**: No significant differences were observed between experimental conditions for TLim or for any physiological or psychophysiological variable. Only main effects of time were detected, reflecting expected exercise-induced responses, with small effect sizes and no evidence of a dose–response relationship across melatonin conditions. Baseline values were comparable among sessions. These findings indicate that acute melatonin administration at doses ranging from 5 to 20 mg does not elicit ergogenic effects nor modulate physiological or psychophysiological responses during prolonged individualized cycling exercise in healthy individuals. **Conclusions**: In male, healthy, physically active individuals inexperienced in cycling, acute melatonin administration at the doses tested did not produce ergogenic effects or alter physiological and psychophysiological responses during prolonged, individualized cycling exercise.

## 1. Introduction

Melatonin (*N*-acetyl-5-methoxytryptamine) is an endogenous hormone that regulates key physiological processes, including circadian rhythms and the sleep–wake cycle [1,2]. Its synthesis starts from tryptophan via sequential conversion to serotonin, *N*-acetylserotonin, and finally melatonin through the actions of key enzymes [3]. This pathway is tightly regulated by the circadian system, with nocturnal norepinephrine release activating adrenergic receptors on pinealocytes, stimulating nighttime melatonin production [4,5,6]. Both melatonin and its metabolites exhibit potent antioxidant, anti-inflammatory, and antitumor properties [7,8]. Moreover, this molecule modulates energy substrate availability and storage, as well as components of the mitochondrial electron transport chain [9,10,11,12], thereby playing a significant role in intermediary metabolism [13,14]. Owing to these biological properties, scientific interest in melatonin has increased substantially, particularly within sports science, where growing attention has been directed toward elucidating its effects on physical exercise and exercise-induced adaptations [15,16,17,18,19,20].

Evidence regarding the ergogenic effects of melatonin on physical exercise remains heterogeneous. One recent systematic review indicates that acute melatonin administration does not consistently enhance traditional performance outcomes such as aerobic capacity, maximal strength, or power output [19]. Further systematic reviews draw similar conclusions for doses from 5 mg to 100 mg, administered before or after exercise [17,21]. However, doses in the lower range (5–10 mg) have been shown to attenuate exercise-induced oxidative stress, muscle damage, and inflammation, and to preserve physical performance during periods of intensive training or following sleep deprivation [22,23,24]. Higher doses (e.g., 100 mg) improved antioxidant capacity and reduced markers of muscle and liver damage, but do not confer additional ergogenic effects on performance [25].

Regarding continuous and prolonged exercise, acute melatonin doses between 3 and 6 mg do not prolong time to exhaustion [15,17,21], but significantly attenuates exercise-induced oxidative stress and muscle damage biomarkers following exhaustive exercise [26]. Complementary evidence from animal models, which typically use pharmacological doses of melatonin ranging from 5 to 10 mg/kg, indicates increased endurance capacity and enhanced tolerance during exhaustive swimming or running protocols, supporting a modulatory role of melatonin on oxidative balance, mitochondrial function, and cellular metabolism under high physiological stress [20,27].

The effects of melatonin on prolonged physical exercise remain controversial, and a dose-dependent approach within this context may help elucidate these inconsistencies. In general, the broader literature indicates that the biological effects of melatonin are dose-dependent, with distinct physiological responses observed across low, physiological, and pharmacological concentrations. At low (sub-physiological) concentrations, melatonin primarily acts as chronobiotic, synchronizing circadian rhythms and promoting sleep onset via receptor activation, without significant systemic effects [1,4]. In physiological concentrations, melatonin regulates the sleep–wake cycle and core body temperature and exerts antioxidant and anti-inflammatory actions, contributing to immune modulation, cardiovascular regulation, and mitochondrial redox homeostasis [7,28,29]. Regarding pharmacological concentrations, melatonin exhibits enhanced antioxidant capacity, attenuates exercise-induced oxidative stress and muscle damage, modulates endocrine function, and may exert neuroprotective and oncostatic effects [4,30]. Despite this well-established dose dependency, the current literature lacks double-blind studies that systematically compare different melatonin doses while specifically examining their ergogenic effects during prolonged physical exercise.

A significant limitation in current sports science research regarding melatonin’s effects on exercise is the failure to account for individual metabolic differences. Most studies employ fixed intensities based on maximal variables, which may result in heterogeneous physiological stress among participants. In contrast, prescribing exercise at a relative intensity based on a well-established physiological phenomenon, such as the Anaerobic Threshold (AnT), ensures that all individuals perform within the same physiological domain, thereby minimizing inter-individual variability in fatigue mechanisms. At submaximal intensities, exercise tolerance is commonly influenced by progressive thermoregulatory strain and central fatigue rather than rapid metabolic acidosis [31,32]. Within this physiological context, a workload corresponding to 80% of the AnT is expected to fall predominantly within the oxidative domain, where perceived exertion and central regulatory mechanisms play a major role in determining time to exhaustion. Although melatonin has been associated with hypothermic and vasodilatory effects [33,34], its functional relevance in this context is expected to manifest primarily through modulation of central autonomic activity and perceptual responses. Through sympatholytic and autonomic-modulating actions [4], melatonin may attenuate the rise in the perceived exertion, a key determinant of exercise tolerance during prolonged submaximal exercise. Additionally, given the predominant reliance on oxidative metabolism at this intensity, melatonin’s mitochondrial-targeted antioxidant properties [12,35] may help preserve electron transport chain efficiency and limit reactive oxygen species accumulation, potentially reducing afferent feedback associated with metabolic stress and contributing to lower perceived effort.

The aim of the present study was to investigate the acute effects of melatonin administration at different doses (i.e 5, 12.5 and 20 mg) on physiological and psychophysiological responses during prolonged cycling exercise performed under controlled and individualized conditions. All selected doses are considered pharmacological in humans, as they exceed typical physiological nocturnal secretion levels [1]. The 5 mg dose was chosen because it represents one of the most used dosages in exercise-based studies and has been shown to attend exercise-induced oxidative stress and muscle damage. The intermediate (12.5 mg) and higher (20 mg) doses were included to explore a potential dose–response relationship, given evidence that melatonin’s antioxidant, mitochondrial, and systemic effects may vary according to concentration. Considering that the sites of action of melatonin may vary as a function of dosage, potentially reflecting the engagement of distinct molecular mechanisms [36], it was hypothesized that melatonin administration would induce dose-dependent modulation of physiological and psychophysiological responses, with a potential fatigue-delaying effect reflected by prolonged time to exhaustion. Given the acute design of the study, the expected effects were primarily related to mechanisms capable of manifesting within a single exercise session. Specifically, melatonin ingestion was hypothesized to modulate autonomic and perceptual responses, potentially attenuating the rise in rating of perceived exertion during acute exercise. Additionally, due to its systemic metabolic and antioxidant properties, melatonin may influence circulating metabolic responses (e.g., lactate and glucose), which could indirectly contribute to exercise tolerance.

## 2. Methods

### 2.1. Participants

Fifteen male volunteers aged 18 to 35 years were recruited through dissemination of the study on social media platforms. All participants received detailed verbal and written information regarding the study objectives, as well as the potential risks and benefits associated with the experimental procedures. After clarification of any questions, those who agreed to participate provided written informed consent by signing the Informed Consent Form, which had been previously approved by the Research Ethics Committee of Universidade São Francisco (CAAE: 70728123.9.0000.5514).

Eligibility for participation required individuals to be classified as physically active, defined as achieving at least 600 MET-min/week according to the International Physical Activity Questionnaire (IPAQ–short version) [37,38]. The IPAQ is a standardized, self-reported instrument used to estimate habitual physical activity levels in adults across different domains and intensities. Participants were also required to have medical clearance for the performance of strenuous physical exercise, to be unfamiliar with cycling-based exercise protocols, and to report no use of exogenous melatonin. Additionally, during the first visit, participants were asked whether their habitual sleep pattern was considered regular and whether they experienced sleep disturbances or excessive daytime sleepiness. Volunteers were excluded if they did not meet the inclusion criteria or if they reported current or continuous use of medications, dietary supplements, anabolic steroids, smoking habits, joint pain or any diagnosed metabolic, cardiovascular, or respiratory disease that could limit or contraindicate the performance of strenuous physical exercise. Additionally, volunteers who reported sleep disturbances—such as regularly waking during the night, not feeling rested upon awakening, or experiencing excessive daytime sleepiness—were excluded from the study.

### 2.2. Experimental Design

**Figure 1:**
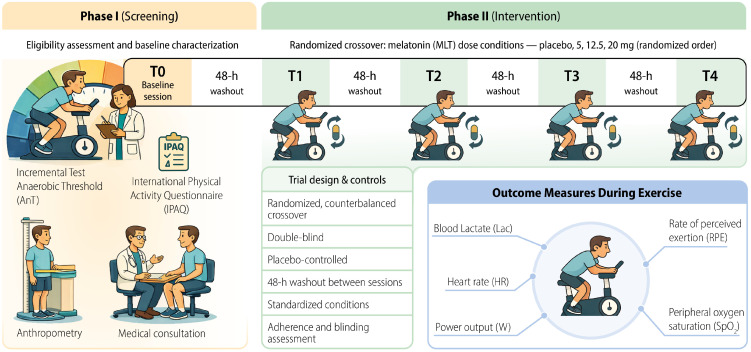
Study design and experimental protocol. Phase I (screening) included eligibility assessment and baseline characterization, comprising anthropometry, medical consultation, the International Physical Activity Questionnaire (IPAQ), and an incremental test to determine the anaerobic threshold. Phase II followed a randomized, double-blind, placebo-controlled, counterbalanced crossover design, in which participants completed exercise sessions under four melatonin (MLT) dose conditions (placebo, 5, 12.5, and 20 mg) administered in randomized order, with a 48 h washout period between sessions. Outcome measures collected during exercise included blood lactate concentration, heart rate, power output, rate of perceived exertion, and peripheral oxygen saturation.

The intervention protocol was systematically described using the Template for Intervention Description and Replication (TIDieR) checklist to support reproducibility in subsequent studies (Supplementary File S1). All procedures were carried out in the Multidisciplinary Research Laboratory at Universidade São Francisco. A temperature-controlled room (22 °C) was designated for the installation of all equipment necessary to ensure safe and controlled execution of the physical exercise protocol. Participants were instructed to maintain their usual dietary and hydration practices and to refrain from strenuous physical activity, as well as from alcohol and caffeine consumption, for at least 96 h prior to the testing sessions. Following confirmation of eligibility, participants completed an initial experimental session in which an incremental exercise protocol was administered to determine the anaerobic threshold intensity (AnT) and the maximal workload attained during the test. In addition to determining the AnT, this test also served as a familiarization session with the ergometer used for the individualized and exhaustive exercise trials. Four additional visits were scheduled, separated by a 48 h washout period (Figure 1). Prior to each session involving exhaustive exercise, with or without melatonin administration, participants were asked whether they had maintained the same regular sleep pattern they reported during the initial visit. Additionally, although participants were instructed to maintain consistent dietary habits throughout the study, this information was reassessed before each exhaustive exercise session, regardless of melatonin supplementation.

This washout period was considered sufficient both to eliminate residual melatonin effects and to ensure adequate recovery between exercise sessions. Exogenous melatonin has a short elimination half-life in humans (approximately 30–60 min for immediate-release formulations), with plasma concentrations typically returning to baseline within a few hours even after pharmacological doses [4,39]. Therefore, the 48 h interval is largely conservative and reduces any risk of carryover. In addition to pharmacological washout, the 48 h interval was considered sufficient to allow full physiological recovery between exhaustive exercise sessions. Evidence from cycling-specific protocols demonstrates that neuromuscular fatigue and performance decrements induced by repeated sprint cycling are fully restored within 48 h, whereas residual impairments may still be present at 24 h [40]. Complementary data from high-intensity functional training models further indicate that metabolic, biochemical, and performance markers return to baseline values within 48 h following strenuous exercise sessions [41]. Although the present protocol involved constant-load cycling rather than repeated sprint or multimodal exercise, these findings support that a 48 h recovery period is sufficient to minimize residual fatigue and prevent carryover effects in physically active individuals undergoing exhaustive efforts.

Participants were randomly allocated to experimental conditions using a computer-generated randomization sequence. The allocation list was created using the online tool Randomizer (www.randomizer.org), which generates sequences based on pseudo-random number algorithms to ensure unbiased and reproducible allocation. The platform allows users to define the number of experimental conditions and participants, automatically distributing assignments in a random order without predictable patterns. Once generated, the sequence is fixed and can be archived, ensuring transparency and reproducibility of the allocation process. To further minimize the risk of selection bias, the randomization procedure was performed by an investigator who was not involved in data collection, exercise supervision, or outcome assessment.

Experimental sessions differed according to the administration of melatonin at varying doses (5, 12.5, and 20 mg) or placebo, and the study was conducted under a double-blind design. All participants were assessed at the same time of day to minimize the influence of circadian rhythm variations on physical performance outcomes. Heart rate (HR), peripheral oxygen saturation (SpO_2_), ratings of perceived exertion (RPE) and blood samples for lactate determination ([Lac]) were obtained before, during (at 3 min intervals, regardless of whether the test was incremental or continuous), and after the exercise tests. Additional blood samples were collected only before and after the exercise tests for blood glucose determination, with no sampling during the exercise bouts to minimize participant discomfort, considering the blood collections already required for lactate analysis.

Participants were asked whether they had obtained adequate sleep on the night preceding each experimental session. The physical efforts were performed on a cycle ergometer (Monark Ergomedic 894 E, Vansbro, Sweden). This apparatus was selected because it allows continuous acquisition of physiological variables without interrupting the exercise task, thereby avoiding potential confounding effects of rest periods on the analyzed outcomes. Saddle height was individually adjusted for each participant based on near-complete knee extension at the lowest point of the pedal cycle. Mechanical variables, including power output and pedaling speed, were continuously monitored using an electronic prototyping system (Arduino, Monza, Italy) integrated with the cycle ergometer. Both the data acquisition and data analysis software were specifically developed for this study using the JupyterLab 3.6.3 (https://jupyter.org/) development environment, with Python version 3.13.2 (https://www.python.org/) employed as the programming language.

### 2.3. Incremental Protocol and Exhaustive Sessions

The incremental protocol was conducted following the methodological procedures adopted by Messias et al., [42]. The test began at an intensity of 75 W, with increments of 27.2 W applied every 3 min stage. Pedaling cadence was fixed at 80 rpm to minimize the potential influence of different cadences on test outcomes [43]. Reaching the predicted maximal heart rate (210 − age × 0.65) [44], inability to maintain the required cadence, or voluntary withdrawal were considered criteria for exhaustion. The method defined as the maximal distance between two end points of the blood lactate concentration vs. power curve (Dmax) [45] was adopted. Therefore, the AnT was determined as the point corresponding to the maximal perpendicular distance between the linear regression and the exponential curve fit. [Lac], HR, SpO_2_, and RPE at these intensities were estimated by linear interpolation.

From a theoretical–practical perspective, exercise performed at a relative intensity corresponding to AnT (100%) should be sustained for at least 30 min. Therefore, all experimental sessions designed to analyze the effects of exogenous melatonin administration were conducted at 80% of the AnT, with a fixed cadence of 80 rpm. Participants exercised until volitional exhaustion, following the same criteria adopted in the incremental protocol. Accordingly, time to exhaustion (TLim) was considered a performance-related outcome.

### 2.4. Melatonin Administration

The order of melatonin administration was randomized, and both participants and investigators responsible for data collection and analysis were blinded to dose allocation. A washout period was implemented between experimental sessions to minimize potential residual effects. Oral melatonin is rapidly absorbed from the gastrointestinal tract, with immediate-release formulations typically reaching peak plasma concentration (Tmax) approximately 30–60 min after ingestion in healthy adults. Reported Tmax values generally range between 30 and 50 min, although inter-individual variability has been described depending on formulation characteristics and metabolic factors [46,47,48,49]. Therefore, a ~30 min interval between melatonin ingestion and exercise onset was adopted to ensure that circulating melatonin levels were rising and approaching peak systemic availability during the exhaustive effort. The selected doses (5, 12.5, and 20 mg) were chosen to reflect a range from commonly used supplemental doses (5 mg or 6 mg), frequently employed in human performance and sleep-related studies, to moderately higher doses (12.5 mg and 20 mg) that have been investigated in experimental and clinical contexts [19]. This range was intended to explore potential dose–response effects within physiologically and clinically relevant boundaries while remaining within safety margins reported in the literature. Placebo capsules were identical in appearance, size, and palatability to the melatonin capsules but contained only inert excipients (Supplementary File S2).

### 2.5. Analysis of Physiological and Psychophysiological Parameters

HR was continuously monitored using a heart rate monitor (POLAR VANTAGE M, Kempele, North Ostrobothnia, Finland), with data stored and subsequently transferred to a computer via the Polar Flow Sync interface. SpO_2_ was assessed using a pulse oximeter (UT-100 Digital MD, Chongqing, China) attached to the participant’s index finger. In addition to data collection, the oximeter served as a safety monitoring tool during the incremental tests, as marked reductions in peripheral oxygen saturation could indicate cyanosis. RPE was evaluated using the Borg [50] scale, where 6 corresponds to “minimal effort” and 20 to “maximal effort”. For [Lac] determination, samples were collected and immediately deposited into 1.5 mL microtubes containing 400 µL of 4% trichloroacetic acid to promote protein precipitation. Lactate concentration was determined using an enzymatic method, with absorbance measured by spectrophotometry at a wavelength of 340 nm. Glucose concentration was determined using the enzymatic colorimetric GOD-PAP method (Glucose Oxidase–Peroxidase–4-Aminophenazone), according to the manufacturer’s instructions (Laborlab, Guarulhos, São Paulo, Brazil). Absorbance was measured at 505 nm using a spectrophotometer. The assay was performed at 37 °C with calibration based on a glucose standard (100 mg/dL), and internal quality controls were applied as recommended by the manufacturer.

### 2.6. Statistical Analysis

A priori sample size estimation was guided by previous randomized, double-blind, placebo-controlled crossover studies that investigated the acute effects of melatonin supplementation on physiological, perceptual, and performance-related responses during exercise. Studies employing repeated-measures designs and ANOVA-based approaches have demonstrated that sample sizes ranging from 10 to 15 participants are sufficient to detect moderate effects of melatonin on physiological and psychophysiological variables, and exercise tolerance when each participant serves as their own control [22,24]. Based on prior methodological literature in exercise science, moderate-to-large effects (Cohen’s d ≈ 0.5–0.8) can be adequately detected with relatively small samples, particularly in within-subject designs, where reduced inter-individual variability enhances statistical power [51]. Considering the within-subject nature of the present protocol, the multiple melatonin doses tested, and the expected moderate effect sizes reported in the literature, a sample of approximately 12–15 participants is expected to provide at least 80% statistical power to detect main effects of treatment and time, as well as dose × time interactions, using two-way repeated-measures ANOVA (α = 0.05).

Statistical procedures were performed using the STATISTICA 7.0 software package (Statsoft, Tulsa, OK, USA). Mean, standard deviation, 95% confidence intervals (CI-(*df* = 14; t = 2.145)), minimum and maximal values were calculated for all variables. Prior to inferential analyses, normality of the data was confirmed by Shapiro–Wilk test. TLim, physiological and psychophysiological parameters were compared using one-way repeated-measures ANOVA. A two-way repeated-measures ANOVA was applied to evaluate the main effects and interactions between melatonin concentrations (between-subjects factor) and time of assessment (pre- and post-exercise; within-subjects factor) on the dependent variables. This approach allowed the investigation not only of the isolated effects of treatment and time, but also whether exercise responses varied according to the administered dose. The assumption of sphericity for repeated-measures comparisons was confirmed using Mauchly’s test. The magnitude of the effect was quantified using partial eta squared, computed from the F statistic and its corresponding numerator and denominator degrees of freedom derived from the repeated-measures ANOVA. In all analyses, the significance level was set at 5%.

The smallest worthwhile change (SWC) for TLim was estimated using a distribution-based approach. Specifically, the SWC was calculated as 0.2 times the between-subject SD of the placebo condition, representing a small effect size according to Cohen’s criteria [52]. Additionally, the typical error (TE) was calculated from the standard deviation of the paired differences between conditions divided by √2, providing an estimate of within-subject variability. The coefficient of variation was expressed as TE divided by the mean performance and multiplied by 100. Standardized effect sizes (Cohen’s d) were computed using the pooled SD and interpreted as small (0.2), moderate (0.5), and large (0.8).

## 3. Results

**Figure 2:**
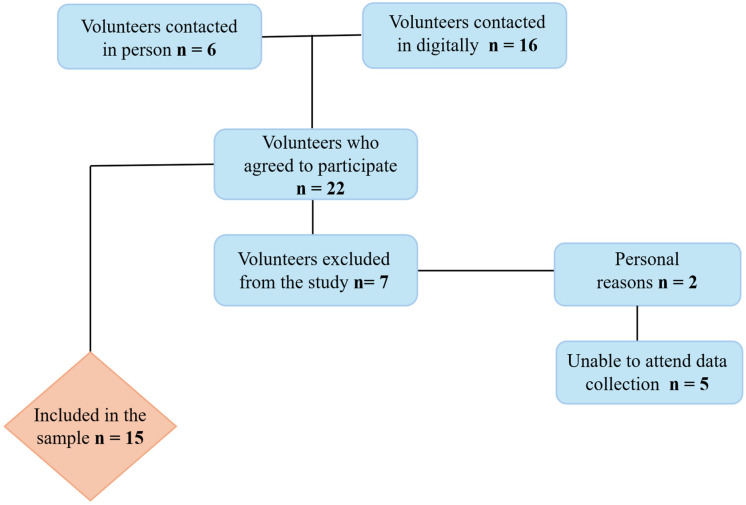
Flowchart illustrating the recruitment process of participants in the present study.

Figure 2 illustrates the flowchart detailing the recruitment process and the number of participants included in this study. Recreational physical activity was reported by the participants, with gym-based exercise being the most prevalent modality (60.0%), followed by recreational running (13.3%). Other leisure activities were less frequent and included judo, jiu-jitsu, volleyball, and basketball, each reported by 6.7% of the participants.

Table 1 presents the sample characterization. According to the inclusion criteria, all participants were classified as physically active based on the IPAQ. Table 2 reports the results obtained from the incremental test.

Baseline values for physiological and psychophysiological variables did not differ between the experimental sessions (Table 3). Further, no significant differences were observed among the experimental sessions for these variables, a finding supported by the low partial eta squared values (Table 4). In terms of mean differences, the placebo session showed higher TLim values compared with melatonin supplementation, with reductions of approximately 8.1%, 7.5%, and 10.3% for the 5 mg, 12.5 mg, and 20 mg doses, respectively. Based on the between-subject SD of the placebo condition (i.e., 1000 s), the SWC was 200 s. The mean differences between placebo and the 5 mg, 12.5 mg, and 20 mg conditions were −175 s, −161 s, and −223 s, respectively.

**Figure 3:**
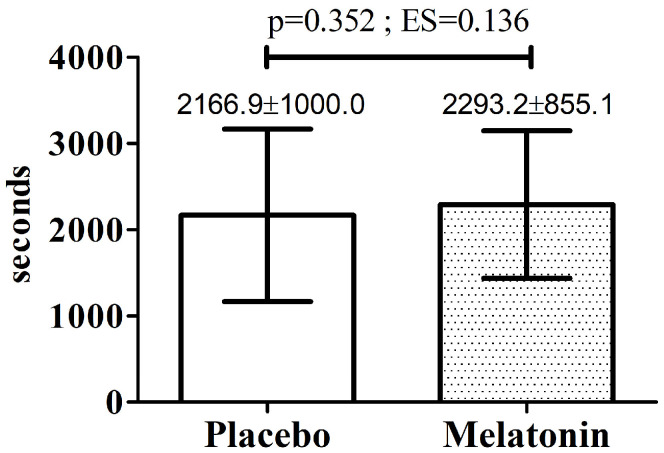
Comparison of time to exhaustion (TLim) between the placebo test and the test showing the best performance with melatonin use, regardless of dose; **ES**—Effect Size. The data are presented as mean and standard deviation.

**Figure 4:**
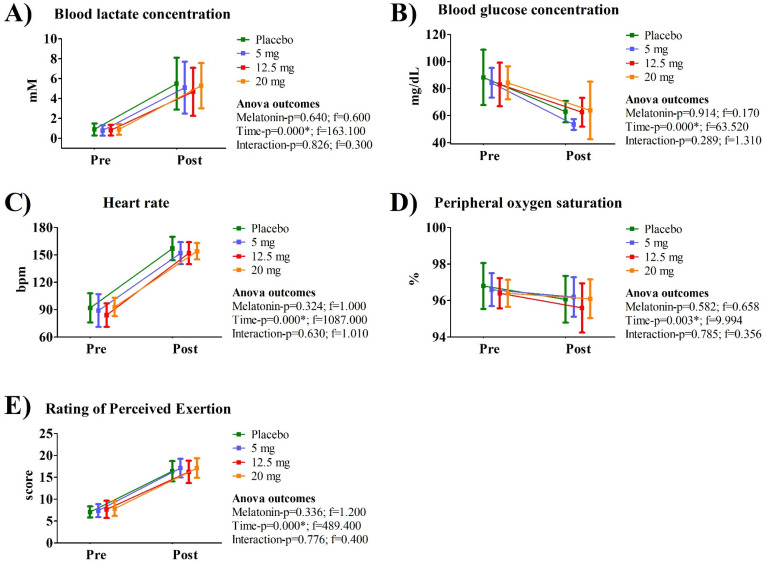
Comparison between pre- and post-session values for physiological and psychophysiological parameters across the experimental sessions. The term “Post” refers to the time point at exhaustion. The data are presented as mean and standard deviation. (**A**) Blood lactate concentration; (**B**) Blood glucose concentration; (**C**) Heart rate; (**D**) Peripheral oxygen saturation; (**E**) Rating of Perceived Exertion; * Indicates a significant effect (*p* ≤ 0.05).

Figure 3 presents a paired comparison between the TLim obtained under the placebo condition and the best performance with melatonin use, regardless of dose. No statistically significant difference was observed in this context. Among the tests evaluated, four volunteers demonstrated better performance with the 5 mg or 12.5 mg dose, whereas seven showed their best performance with the 20 mg dose (Supplementary File S3). Figure 4 shows the comparison between pre- and post-session values for physiological and psychophysiological parameters across the experimental sessions. Significant effects were observed only for time. Mean values, standard deviations, and 95% confidence intervals for the data presented in Figure 3 and Figure 4 are provided in Supplementary File S4.

## 4. Discussion

Our initial hypotheses were not supported. Acute melatonin administration at the tested doses failed to elicit ergogenic effects or to modify physiological and psychophysiological responses during exhaustive exercise in physically active individuals but unexperienced in cycling.

Evidence regarding the ergogenic effects of melatonin during cycling exercise remains limited and inconsistent. In double-blind, randomized, crossover studies involving trained male cyclists, acute administration of 5 mg of melatonin 15 min before laboratory-simulated cycling time trials (32.2 km) did not improve performance outcomes under thermoneutral conditions [53]. Similarly, Atkinson, Jones [33] reported that 5 mg of melatonin ingested during the daytime did not enhance performance during a short-duration 4 km cycling time trial, despite reductions in alertness and heart rate. In contrast, Beck, Messias [16] demonstrated that acute administration of 6 mg of melatonin enhanced aerobic tolerance and was accompanied by favorable biochemical and hematological responses, suggesting that melatonin may preferentially influence exercise tolerance rather than time-trial or maximal performance.

The present study advances previous reports by systematically testing multiple melatonin doses within a within-subjects design, minimizing inter-individual variability. The optimal dose of melatonin remains a topic of active scientific debate, particularly in the context of exercise, as prior studies have employed heterogeneous dosing strategies across different exercise modalities. Systematic reviews specifically addressing melatonin supplementation in the context of exercise highlight a wide and poorly standardized range of administered doses, which represents a major limitation in the current literature. The systematic review by Faria, Messias [19] reported that acute oral melatonin doses used in human performance studies vary markedly, typically ranging from 5 to 100 mg, with no clear dose–response relationship for ergogenic outcomes. Likewise, San-Miguel, [21] demonstrated substantial variability in dosing protocols across randomized controlled trials, including low (≤5 mg), moderate (5–10 mg), and high (≥20 mg) doses, administered either acutely or over short supplementation periods. Importantly, both reviews emphasized that inconsistent dosing strategies hinder direct comparisons between studies and may partly explain the contradictory findings regarding performance enhancement, underscoring the need for controlled, within-subject dose–response investigations. Collectively, these findings indicate that the effects of melatonin on cycling performance are highly context-dependent and may vary according to dose, timing of ingestion, exercise protocol, and the specific performance outcome assessed.

When considering the magnitude and direction of the observed differences, the placebo condition consistently demonstrated higher mean TLim values compared to all melatonin doses. Although the reductions (−161 to −223 s) did not exceed the SWC in all comparisons, the uniform direction of effect suggests a potential physiological influence. Melatonin is well established as a chronobiotic hormone with soporific properties [54,55,56], and acute daytime administration has been shown to reduce alertness [57], core body temperature [58], and psychomotor performance [33]. Furthermore, integrative evidence indicates that nutritional strategies may interact with physical activity to influence psychological well-being, cognitive performance, and perceived effort in university-aged populations [59]. Experimental studies indicate that exogenous melatonin administered outside the endogenous nocturnal peak may acutely increase subjective sleepiness and reduce vigilance [60,61]. Therefore, although no statistically significant impairment was detected, the consistent trend toward lower TLim under melatonin supplementation may reflect subtle reductions in arousal state during daytime exercise. This perspective, however, warrants further investigation.

Several methodological and physiological factors inherent to the present protocol may explain the lack of ergogenic effects of acute melatonin administration. Although participants were classified as physically active, they were deliberately selected to be inexperienced in cycling to minimize training-specific adaptations [62]. Under this condition, performance during exhaustive cycling is likely limited primarily by task-specific neuromuscular coordination and local muscular fatigue, rather than by central regulatory or metabolic processes, which are the pathways more plausibly influenced by this hormone [1,4,9,13,17]. Consequently, even if melatonin exerted subtle central or systemic effects, these may have been overridden by peripheral, skill-related constraints, reducing the likelihood of detecting an ergogenic response in time-to-exhaustion outcomes.

Despite the use of multiple doses, inter-individual variability in melatonin pharmacokinetics may have attenuated potential dose–response effects. Substantial variability in oral absorption and plasma Tmax are reported even with immediate-release formulations [47]. Likewise, inter-individual differences in maximal concentration following oral melatonin are reported [63], suggesting that optimal systemic concentrations at the time of peak exercise demand may not be reliably attained in all participants. This factor helps explain the contrasting results observed with melatonin in animals subjected to exercise. Reports in rodents have demonstrated that acute intraperitoneal administration of melatonin at doses of approximately 10 mg·kg^−1^ significantly improved endurance outcomes, such as time to exhaustion during exhaustive swimming or treadmill running and modulated metabolic and oxidative stress markers [18,20,27]. These effects are likely facilitated by the high systemic exposure achieved via injectable routes, which bypass gastrointestinal absorption and first-pass metabolism, resulting in more predictable pharmacokinetics compared with oral administration in humans. While such preclinical findings provide mechanistic support for potential ergogenic benefits of melatonin, translation to humans is complicated by inter-individual variability in oral bioavailability, differences in metabolic rate, and lower relative doses typically used in human trials, which may explain the absence of detectable performance effects in the present study.

Although no ergogenic effect was observed, a potential explanation lies in changes in the physiological and psychophysiological responses that were assessed. Previous reports also demonstrated that melatonin does not modulate HR [33], lactate, glucose [22,64] or perceived exertion [53] during exercise. The absence of modulation in these responses may be attributed to the context-dependent nature of melatonin’s physiological actions, which include hypothermic, anxiolytic, and sympatholytic effects [65,66,67,68,69], that may blunt cardiovascular and metabolic activation during high-intensity exercise performed in thermoneutral conditions. Moreover, melatonin’s antioxidant and anti-inflammatory properties [25,70,71]—often proposed as key mediators of exercise-related benefits—are more likely to exert measurable effects during prolonged exercise, repeated bouts, or recovery phases rather than during a single acute exhaustive task. In this sense, individual differences in circadian phase and chronotype may have further influenced responsiveness to daytime melatonin administration, potentially masking subtle physiological or psychophysiological effects at the group level.

Another important aspect to consider is the distinction between acute and chronic melatonin administration in the context of physical performance. Acute melatonin ingestion (typically a single dose administered 30–60 min before exercise or sleep) primarily affects immediate outcomes, including sleep quality, next-day psychomotor and physical performance, and short-term recovery from exercise-induced oxidative stress and muscle damage. For instance, acute doses of 6–10 mg have been shown to improve maximal performance, reaction time, and attenuate fatigue and muscle damage on the following day, particularly after sleep deprivation or strenuous late-evening exercise [22,24,72]. However, acute supplementation does not consistently enhance aerobic or strength performance across all exercise contexts [17,53]. In contrast, chronic melatonin supplementation (daily intake for days to weeks, typically at bedtime) appears to promote cumulative adaptations, such as sustained reductions in oxidative stress and muscle or liver damage, alongside improved recovery during intensive training periods. Studies administering 5–100 mg nightly for 6 days to 4 weeks in athletic populations report attenuation of cellular damage, enhanced antioxidant enzyme activity, and preservation of performance under repeated or prolonged training stress [23,25,73]. Collectively, these findings suggest that chronic supplementation strategies may be more relevant for long-term adaptation and recovery, whereas acute administration is less consistently associated with direct ergogenic effects, which is consistent with the findings of the present study.

It is also important to consider the ecological relevance of the performance model adopted. TLim tests, although highly sensitive to physiological perturbations and commonly used to detect subtle experimental effects, differ conceptually from self-paced time-trial paradigms, which more closely replicate real-world competitive settings. Time-trial performance integrates pacing strategy, decision-making, and motivational components, thereby enhancing ecological validity, whereas constant-load TLim protocols primarily assess tolerance to a fixed physiological strain. Therefore, the absence of ergogenic effects observed in the present study should be interpreted within the context of a controlled time-to-exhaustion model. Future double-blind, crossover investigations employing time-trial designs may help clarify whether melatonin differentially influences performance under conditions that more closely reflect competitive environments.

This study presents strengths that add novelty and robustness to the current literature on melatonin and exercise performance. To the best of our knowledge, this is the first investigation to systematically examine the acute effects of multiple melatonin doses under individualized exercise sessions. The within-session design, combined with individualized workload prescription, enhanced internal validity by minimizing interindividual variability and ensuring comparable physiological strain across experimental conditions. Additionally, the simultaneous assessment of performance outcomes alongside physiological and psychophysiological markers provides an integrated view of melatonin’s potential mechanisms of action during exhaustive exercise.

Despite the strengths, some limitations should be acknowledged when interpreting the present findings. This report focused on male, healthy, physically active individuals, which may restrict the generalizability of the results to females, clinical populations, elite athletes, or individuals with sleep or circadian disturbances. Additionally, direct measures of central nervous system activity, autonomic modulation, or substrate oxidation were not included, limiting mechanistic inferences regarding the pathways through which melatonin might influence exercise tolerance. Further, circulating melatonin concentrations were not measured; therefore, we cannot confirm the pharmacokinetic response or interindividual variability in absorption following supplementation. Not less important, physiological markers potentially sensitive to melatonin administration, such as core body temperature and heart rate variability, were not assessed, limiting our ability to explore mechanistic pathways related to autonomic modulation or thermoregulatory responses. Although the initial session included an incremental test that may have provided a degree of familiarization with the exercise protocol, and the crossover design reduces between-subject variability, the possibility of a residual learning effect across repeated time-to-exhaustion trials cannot be entirely excluded. Such an effect may have influenced performance outcomes despite standardized procedures and randomization of conditions. Importantly, these limitations do not undermine the internal consistency of the findings but rather define the scope within which the conclusions should be interpreted. Future studies should focus on repeated melatonin administration under conditions of circadian stress, integrating autonomic and metabolic assessments, and accounting for individual variability such as chronotype. Extending this approach to trained or clinical populations may better define the contexts in which melatonin is functionally relevant to exercise performance.

## 5. Conclusions

In male, healthy, physically active individuals inexperienced in cycling, acute melatonin administration at the doses tested did not produce ergogenic effects or alter physiological and psychophysiological responses during prolonged, individualized cycling exercise.

## Figures and Tables

**Table 1:** Characterization of the sample included in this study.

N = 15	Mean ± SD	CI (95%)	Min–Max
Age (years)	25 ± 4	23.07–27.20	19–34
Body Mass (kg)	81.84 ± 16.24	73.62–90.07	62.00–110.80
Height (cm)	177.90 ± 7.99	172.95–181.05	167.0–195.0
BMI (km/m^2^)	25.78 ± 4.22	23.65–27.92	20.48–33.23
IPAQ (score)	3343.5 ± 3135.44	1.756–4.930	636–3434

**SD**—Standard Deviation; **CI**—Confidence Interval; **BMI**—Body Mass Index; **IPAQ**—International Physical Activity Questionnaire. The terms **Min** and **Max** refer to the minimum and maximum values, respectively.

**Table 2:** Results derived from the incremental test performed during the first experimental session.

N = 15	Mean ± SD	CI (95%)	Min–Max
TLim (s)	818.06 ± 234	688.48–947.64	582–1368
AnT (W)	126.10 ± 24.98	112.27–139.93	107.00–183.00
[Lac]_Rest_ (mM)	1.012 ± 0.86	0.54–1.49	0.15–2.84
[Lac]_Mean_ (mM)	5.07 ± 1.67	4.11–5.89	2.64–8.30
[Lac]_Exhaustion_ (mM)	8.20 ± 1.95	7.12–9.28	4.84–11.94
[Lac]_AnT_ (mM)	4.55 ± 1.13	3.42–5.68	3.23–6.70
HR_Rest_ (bpm)	91 ± 17	81–100	67–128
HR_Mean_ (bpm)	154 ± 12	147–160	128–175
HR_Exhaustion_ (bpm)	178 ± 11	171–184	163–197
HR_AnT_ (bpm)	151 ± 10	141–161	130–163
SpO_2Rest_ (%)	96.4 ± 0.9	95.9–96.9	94.0–98.0
SpO_2Mean_ (%)	95.9 ± 1.5	95.0–96.7	91.8–97.7
SpO_2Exhaustion_ (%)	96.1 ± 1.5	95.2–96.9	94.0–99.0
SpO_2AnT_ (%)	96.0 ± 0.9	95.1–96.9	94.3–97.2
RPE_Rest_ (score)	7.0 ± 1.3	6.2–7.72	6–10
RPE_Mean_ (score)	13.2 ± 1.7	12.2–14.1	11–17
RPE_Exhaustion_ (score)	17.1 ± 1.8	16.1–18.1	14–20
RPE_AnT_ (score)	13.9 ± 1.9	12.0–15.8	11.2–17.6

**TLim**—Time to Exhaustion; **SD**—Standard Deviation; **CI**—Confidence Interval; **AnT**—Anaerobic Threshold Intensity; **[Lac]**—Blood Lactate Concentration; **HR**—Heart Rate; **SpO_2_**—Peripheral Oxygen Saturation; **RPE**—Rating of Perceived Exertion. The terms **Min** and **Max** refer to the minimum and maximum values, respectively.

**Table 3:** Within-session comparison of baseline values across experimental conditions.

N = 15	Placebo	Melatonin (5 mg)	Melatonin (12.5 mg)	Melatonin (20 mg)	One-Way Anova	Partial Eta Squared
[Lac]_Rest_ (mM)	0.96 ± 0.64 _(0.55–1.25)_	0.79 ± 0.51 _(0.51–1.07)_	0.83 ± 0.53 _(0.54–1.12)_	0.90 ± 0.53 _(0.61–1.19)_	*p* = 0.377 f = 1.057	0.513
HR_Rest_ (bpm)	92 ± 16 _(83–100)_	89 ± 18 _(79–99)_	84 ± 13 _(76–91)_	93 ± 10 _(87–98)_	*p* = 0.126 f = 1.505	0.600
SpO_2Rest_ (%)	96.8 ± 1.2 _(96.1–97.5)_	96.6 ± 0.9 _(96.1–97.1)_	96.4 ± 0.8 _(96.0–96.8)_	96.4 ± 0.7 _(96.0–96.8)_	*p* = 0.608 f = 0.615	0.380
RPE_Rest_ (score)	7.1 ± 1.2 _(6.4–7.8)_	7.4 ± 1.5 _(6.6–8.2)_	7.7 ± 1.9 _(6.6–8.8)_	7.8 ± 1.0 _(7.2–8.4)_	*p* = 0.249 f = 1.422	0.587

**[Lac]**—Blood Lactate Concentration; **HR**—Heart Rate; **SpO_2_**—Peripheral Oxygen Saturation; **RPE**—Rating of Perceived Exertion. Values in parentheses indicate the 95% confidence interval.

**Table 4:** Within-session comparison of performance outcomes and mean values.

N = 15	Placebo	Melatonin (5 mg)	Melatonin (12.5 mg)	Melatonin (20 mg)	One-Way Anova	Partial Eta Squared
TLim (s)	2166.9 ± 1000.0 _(1613.1–2720.6)_	1991.6 ± 868.7 _(1510.5–2472.7)_	2005.4 ± 748.7 _(1590.7–2420.1)_	1944.2 ± 885.1 _(1454.0–2434.4)_	*p* = 0.587 f = 0.650	0.393
[Lac]_Mean_ (mM)	4.35 ± 1.99 _(3.25–5.45)_	4.20 ± 2.00 _(3.09–5.31)_	4.22 ± 1.76 _(3.25–5.20)_	4.13 ± 1.52 _(3.29–4.97)_	*p* = 0.829 f = 0.294	0.227
HR_Mean_ (bpm)	147 ± 12 _(140–153)_	144 ± 14 _(136–151)_	143 ± 11 _(136–149)_	145 ± 12 _(138–151)_	*p* = 0.477 f = 0.845	0.457
SpO_2Mean_ (%)	96.3 ± 0.9 _(95.5–96.8)_	96.2 ± 0.8 _(95.8–96.6)_	95.8 ± 0.6 _(95.5–96.1)_	96.0 ± 0.7 _(95.6–96.4)_	*p* = 0.561 f = 0.771	0.435
RPE_Mean_ (score)	13.4 ± 1.5 _(12.6–14.2)_	13.7 ± 1.2 _(13.0–14.4)_	13.3 ± 1.6 _(12.4–14.2)_	13.6 ± 1.1 _(13.0–14.2)_	*p* = 0.558 f = 0.698	0.411

**TLim**—Time to Exhaustion; **[Lac]**—Blood Lactate Concentration; **HR**—Heart Rate; **SpO_2_**—Peripheral Oxygen Saturation; **RPE**—Rating of Perceived Exertion. Values in parentheses indicate the 95% confidence interval.

## Data Availability

The full protocol and statistical analysis plan are available upon request to the corresponding author. The data are not publicly available due to ethical restrictions and compliance with Brazil’s General Data Protection Law (LGPD).

## Supplementary Materials

The following supporting information can be downloaded at: https://www.mdpi.com/article/10.3390/nu18050798/s1, File S1: TIDieR Reporting Guideline; File S2: Melatonin–Technical Formulation Information; File S3: Individual comparisons of TLim; File S4: Supplementary tables containing the data from Figure 3 and Figure 4.
