# Supplementary material for: Acute Effects of Different Melatonin Doses on Performance and Psychophysiological Responses During Exhaustive Cycling Exercise: A Double-Blind Crossover Study
Source: Nutrients. 2026 Feb 28;18(5):798. doi: 10.3390/nu18050798 (PMC12987325; doi:10.3390/nu18050798)
Supplement: Supplementary file 1 [file nutrients-18-00798-s001.zip › Supplementary File 1.pdf]

Supplementary

Supplementary File 1: TIDieR Reporting Guideline

Larissa de Castro Pedroso[1], Maria Clara dos Reis[1], Vanessa Bertolucci[1], Luana Alves Silva[1], Ivan Gustavo Masselli dos Reis[1], Wladimir Rafael Beck[2], Pedro Paulo Menezes Scariot[1]; Leonardo Henrique Dalcheco Messias[1].

[1] Research Group on Technology Applied to Exercise Physiology—GTAFE, Health Sciences and Health Data Science Postgraduate Programs, São Francisco University (USF), Bragança Paulista 12916-900, SP, Brazil; larissa.pedroso@mail.usf.edu.br; maria.clara.reis@mail.usf.edu.br; vanessa.bertolucci@mail.usf.edu.br; silva.alves.luana@mail.usf.edu.br; ivan.reis@usf.edu.br; pedro.scariot@mail.usf.edu.br.

[2] Laboratory of Endocrine Physiology and Physical Exercise, Department of Physiological Sciences, Federal University of São Carlos, Washington Luis, Km 235, São Carlos, São Paulo, 13565-905, Brazil; beckwr@ufscar.br.

* Correspondence: leonardo.messias@usf.edu.br

TIDieR
Template for Intervention Description and Replication

The TIDieR (Template for Intervention Description and Replication) Checklist*:

Information to include when describing an intervention and the location of the information

Item	TIDieR Element	Manuscript Location	Complete Description
**1**	BRIEF NAME	Title, Abstract (Page 1)	Acute oral melatonin supplementation at different doses (5 mg, 12.5 mg, and 20 mg) administered prior to exhaustive cycling exercise in a randomized, double-blind, placebo-controlled, counterbalanced crossover design.
**2**	WHY (Rationale, Theory, Goal)	Introduction (Pages 2–4); Discussion (Pages 12–14)	Melatonin is a pleiotropic hormone with antioxidant, mitochondrial, metabolic, and chronobiotic properties that may influence exercise performance and psychophysiological responses. However, evidence regarding its acute, dose–response effects during high-intensity endurance

Academic Editor: Firstname Lastname

Received: date
Revised: date
Accepted: date
Published: date

Copyright: © 2026 by the authors. Submitted for possible open access publication under the terms and conditions of the Creative Commons Attribution (CC BY) license.

			exercise in humans is limited. The goal of the intervention was to examine whether different acute doses of melatonin modulate performance (time to exhaustion) and physiological and perceptual responses during exhaustive cycling exercise.
**3**	WHAT - Materials	Methods (Page 5, Section 2.3)	Melatonin and placebo were provided in identical opaque capsules. Melatonin doses were 5 mg, 12.5 mg, and 20 mg. Placebo capsules contained inert excipients with identical appearance and taste. Capsules were prepared, coded, and stored by an independent researcher not involved in data collection or analysis to ensure blinding.
**4**	WHAT - Procedures	Methods (Pages 5–6, Sections 2.4–2.5); Figure 1	Participants ingested one capsule (placebo or melatonin) approximately 30 minutes before exercise. Each participant completed four experimental sessions (placebo, 5 mg, 12.5 mg, and 20 mg) in randomized order, separated by a 48-hour washout period. After supplementation, participants performed continuous cycling exercise at 80% of the anaerobic threshold until voluntary exhaustion.
**5**	WHO PROVIDED	Methods (Pages 4–5, Sections 2.1–2.2)	The intervention was delivered by researchers with expertise in exercise physiology. Capsule preparation and coding were performed by an independent investigator. Exercise testing and monitoring were conducted by trained laboratory staff under standardized conditions.
**6**	HOW - Delivery Mode	Methods (Pages 5–6, Sections 2.3–2.5)	Oral administration of a single capsule under laboratory conditions, followed by supervised exercise testing. Supplement ingestion was unsupervised beyond confirmation of intake prior to exercise, and the intervention was delivered individually.
**7**	WHERE - Location	Methods (Page 4, Section 2.2)	All experimental sessions were conducted in the Exercise Physiology Laboratory of Universidade São Francisco, Bragança Paulista, SP, Brazil, under controlled environmental conditions.
**8**	WHEN and HOW MUCH - Dosing Schedule	Methods (Pages 5–6, Sections 2.3–2.5)	A single acute dose of melatonin (5 mg, 12.5 mg, or 20 mg) or placebo was administered approximately 30 minutes before exercise. Each condition was tested once per participant, with a 48-hour washout period between sessions.
**9**	TAILORING - Personalization	N/A	The intervention was not tailored or personalized. All participants received the same supplementation doses and followed

			the same exercise protocol across experimental sessions.
**10**	MODIFICATIONS	N/A	No modifications to the intervention protocol were made during the study.
**11**	HOW WELL - Planned	Methods (Page 6, Section 2.6)	Intervention fidelity was ensured by standardized capsule preparation, double-blinding, controlled timing of supplementation, and identical exercise protocols across all experimental conditions.
**12**	HOW WELL - Actual	Results (Pages 8–9, Section 3)	All participants completed the four experimental conditions as planned. No deviations from the supplementation protocol or exercise procedures were reported.

Disclaimer/Publisher's Note: The statements, opinions and data contained in all publications are solely those of the individual author(s) and contributor(s) and not of MDPI and/or the editor(s). MDPI and/or the editor(s) disclaim responsibility for any injury to people or property resulting from any ideas, methods, instructions or products referred to in the content.
