# Supplementary material for: Acute Effects of Different Melatonin Doses on Performance and Psychophysiological Responses During Exhaustive Cycling Exercise: A Double-Blind Crossover Study
Source: Nutrients. 2026 Feb 28;18(5):798. doi: 10.3390/nu18050798 (PMC12987325; doi:10.3390/nu18050798)
Supplement: Supplementary file 1 [file nutrients-18-00798-s001.zip › Supplementary File 2.pdf]

Alquimia
Farmácia de Manipulação

CNPJ. 00.421.256/0001-72 INSCR. EST. 225.092.443.119

Bragança Paulista, 01 de Junho de 2025

Ao

Programa de Pós-Graduação Stricto Sensu em Ciências da Saúde da Universidade São Francisco

Dr. Leonardo Henrique Dalcheco Messias - Docente em Fisiologia

Declaração

Conforme solicitado, desde então colaboramos com a Universidade São Francisco para realizar o estudo com a referida Melatonina, a Alquimia farmácia de manipulação teve o prazer de fornecer a Melatonia que ressalta a referida matéria prima que está licenciado pelos órgãos exigentes da ANVISA e que atende as referências internacionais de uso farmacêutico.

Em anexo segue uma cópia do laudo da matéria prima utilizada no estudo ocorrido.

Sem mais,

Atenciosamente,

Silmara Serinoli Rodrigues
CRF 14309

R. Dr. Candido Rodrigues, 140 - CEP 12.900-360 - Fone (11) 4033-4129
Centro – Bragança Paulista - SP www.alquimianet.com.br

140597

LEPUGE

CERTIFICADO DE ANÁLISE

INFORMAÇÕES DO PRODUTO	
Produto..: MELATONINA	Código..: 531900.000050
Origem... CHINA	Procedência..: BRASIL
Lote Interno..: AUTO027745	Lote do Fabricante..: 20230801
Fabricação: 18/08/2023	Validade: 17/08/2026
Peso Molecular: 232.28	Formula Molecular: C13H16N202
DCB: 11038	CAS: 73-31-4
Categoria Terapeutica: SUPLEMENTO ALIMENTAR	Parte Utilizada: Não Aplicável
Nome Científico: Não Aplicável	Familia: Não Aplicável
Ordem de Produção: Não Aplicável	Data de Análise: 09/02/2024
Data de Emissão: 14/02/2024	Nota Fiscal: 000266638

Análises

Ensaio	Especificação	Resultado
ARSENIO	MÁXIMO 1 PPM	<1 PPM
ASPECTO FISICO	PÓ-CRISTALINO BRANCO OU QUASE BRANCO	PO CRISTALINO QUASE BRANCO
CADMIO	MÁXIMO 1 PPM	DE ACORDO
CHUMBO	MÁXIMO 1 PPM	DE ACORDO
CLORETOS	MÁXIMO 0.02%	0,02%
COMPOSTOS RELATADOS	IMPUREZA INDIVIDUAL, MÁXIMO 0,1%	0.040%
COMPOSTOS RELACIONADOS	IMPUREZA TOTAL MÁXIMO 1,0%	6.197%
IDENTIFICAÇÃO - IR	O ESPECTRO DA AMOSTRA CORRESPONDE AO DO PADRÃO	DE ACORDO
IDENTIFICAÇÃO - UV	ABSORTIVIDADE CALCULADO EM BASE ANIDRA, NAD DIFERE MAIS QUE 3.0%	DE ACORDO
IDENTIFICAÇÃO - HPLC	O PRINCIPAL PICO DO CROMATOGRAMA OBTIDO COM SOLUÇÃO TESTE CORRESPONDE AO PRINCIPAL PICO DO CROMATOGRAMA OBTIDO COM A SOLUÇÃO PADRAO	DE ACORDO
MERCÚRIO	1 MÁXIMO 0.1 PPM	DE ACORDO
SOLUBILIDADE	SOLÚVEL EM ETANOL, POUCO SOLÚVEL EM ÁGUA	DE ACORDO
DENSIDADE APARENTE	INFORMATIVO	0.3785 G/ML
RESÍDUO POR INCINERAÇÃO	MAX. 0.10%	0.33%
PERDA POR SECAGEM (%)	MAX. 1.0%	0.14%
DOSEAMENTO	08.5% A 101.5%	99,40%

REFERÊNCIAS / OBSERVAÇÕES

1. DADOS RESULTANTES DA AVALIACAO ANALITICA REALIZADA PELO FABRICANTE/ FORNECEDOR (USP-NF CURRENT DOCID: GUID-454646BE-F1DF-458C-9011-1FBBCCEFE5BC_4_EN-US).
2. SOLUBILIDADE EM AGUA COM AGITACAO.
3. ARSENIO TESTE REALIZADO DE ACORDO COM ORIENTACAO DO FABRICANTE.
4. CADMIO, MERCURIO, CHUMBO, RESULTADO TRANSCRITO DO LAUDO DE ANALISE DO FABRICANTE (USP 43).
5. FATOR DE CORRECAO: 1,0060.
