# Supplementary material for: Acute Effects of Different Melatonin Doses on Performance and Psychophysiological Responses During Exhaustive Cycling Exercise: A Double-Blind Crossover Study
Source: Nutrients. 2026 Feb 28;18(5):798. doi: 10.3390/nu18050798 (PMC12987325; doi:10.3390/nu18050798)
Supplement: Supplementary file 1 [file nutrients-18-00798-s001.zip › Supplementary File 4.pdf]

Supplementary File 4.

Mean, standard deviation (SD), and 95% confidence intervals (CI) for the comparison of time to exhaustion (TLim) between the placebo trial and the trial demonstrating the best performance following melatonin administration, regardless of dose. The comparisons are presented in Figure 3 of the manuscript.

	Placebo	Melatonin
**Mean**	2166.9	2293.2
**SD**	1000.0	855.1
**CI**	1613.2 – 2720.6	1819.7 – 2766.7

Means, standard deviations, and 95% confidence intervals for the comparison between pre- and post-session values of physiological and psychophysiological parameters across the experimental sessions, as presented in Figure 4 of the manuscript.

N=15	Placebo		Melatonin (5 mg)		Melatonin (12.5 mg)		Melatonin (20 mg)	
	Pre	Post	Pre	Post	Pre	Post	Pre	Post
**[Lac] (mM)**	0.90 ± 0.60 (0.57 – 1.23)	5.50 ± 2.60 (4.06 – 6.94)	0.79 ± 0.51 (0.51 – 1.07)	5.10 ± 2.60 (3.66 – 6.54)	0.83 ± 0.53 (0.54 – 1.12)	4.68 ± 2.42 (3.34 – 6.02)	0.90 ± 0.53 (0.61 – 1.19)	5.29 ± 2.27 (4.03 – 6.55)
**[Glc] (mg/dL)**	88.27 ± 20.42 (76.96 – 99.58)	62.96 ± 7.90 (58.59 – 67.33)	84.27 ± 10.96 (78.20 – 90.34)	53.47 ± 4.00 (51.26 – 55.68)	83.10 ± 16.06 (74.20 – 92.00)	62.49 ± 10.64 (56.60 – 68.38)	84.27 ± 12.18 (77.53 – 91.01)	63.84 ± 21.32 (52.03 – 75.65)
**HR (bpm)**	92 ± 16 (83 – 100)	157 ± 13 (149 – 164)	89 ± 18 (79 – 98)	152 ± 13 (144 – 159)	84 ± 13 (76 – 91)	152 ± 12 (145 – 158)	93 ± 10 (87 – 98)	154 ± 9 (149 – 158)
**SpO_{2Mean} (%)**	96.8 ± 1.2 (96.1 – 97.4)	96.1 ± 1.2 (95.4 – 96.76)	96.6 ± 0.9 (96.10 – 97.10)	96.2 ± 1.1 (95.5 – 96.8)	96.4 ± 0.8 (95.9 – 96.8)	95.6 ± 1.3 (94.8 – 96.3)	96.4 ± 0.7 (96.0 – 96.7)	96.1 ± 1.1 (95.4 – 96.7)
**RPE_{Mean} (score)**	7.1 ± 1.2 (6.4 – 7.7)	16.4 ± 2.3 (15.1 – 17.6)	7.4 ± 1.5 (6.5 – 8.2)	17.1 ± 2.1 (15.9 – 18.2)	7.7 ± 1.9 (6.6 – 8.7)	16.2 ± 2.5 (14.8 – 17.5)	7.8 ± 1.6 (6.9 – 8.6)	17.1 ± 2.2 (15.8 – 18.32)
