# Supplementary figures and images for: Acute Effects of Different Melatonin Doses on Performance and Psychophysiological Responses During Exhaustive Cycling Exercise: A Double-Blind Crossover Study
Source: Nutrients. 2026 Feb 28;18(5):798. doi: 10.3390/nu18050798 (PMC12987325; doi:10.3390/nu18050798)

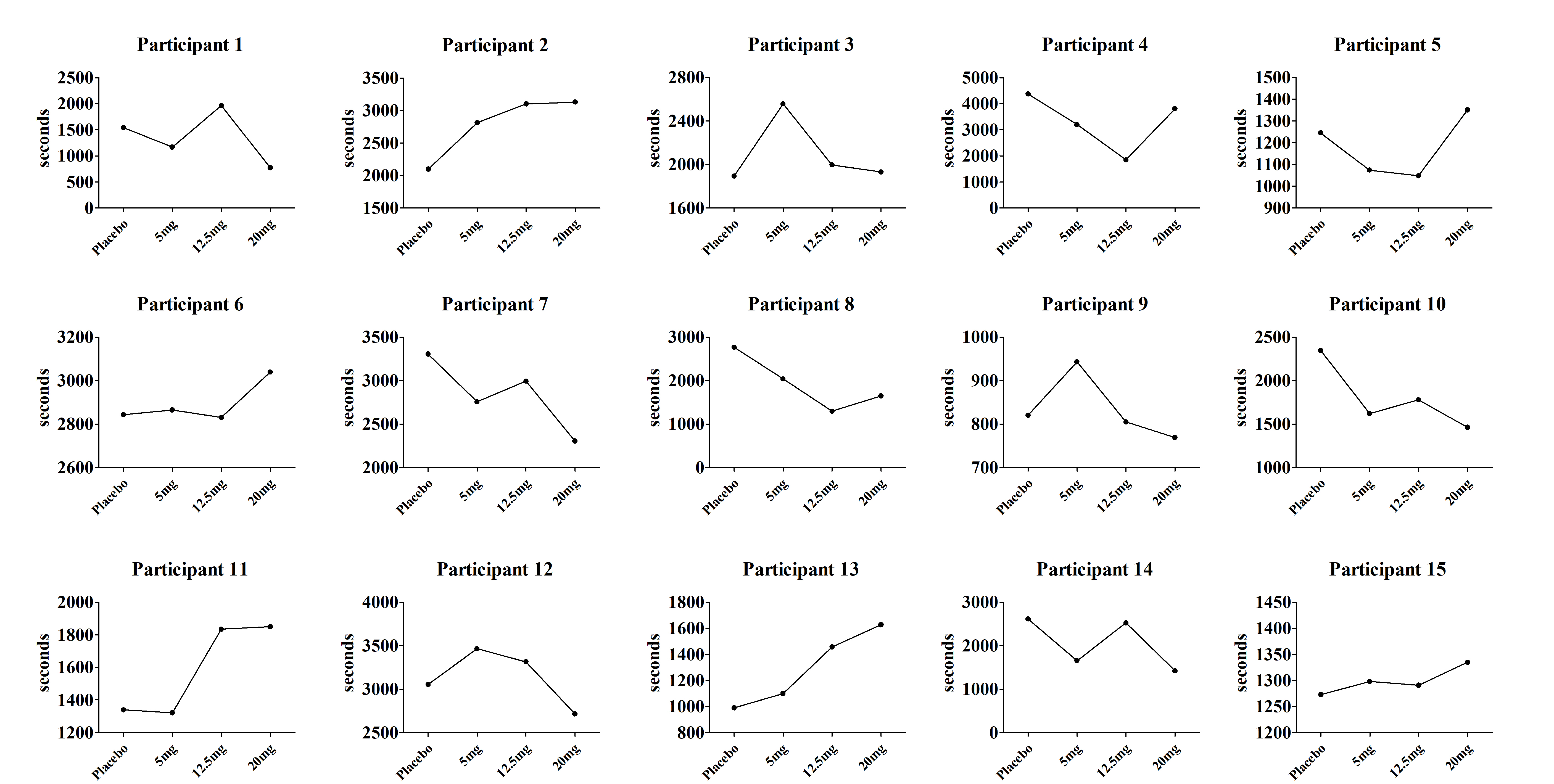

Supplement: Supplementary file 1 [file nutrients-18-00798-s001.zip › Supplementary File 3.jpg]
